# Validity and Reliability of Criteria for Plantar Sensation Assessment Using Semmes–Weinstein Monofilament as a Clinically Usable Index

**DOI:** 10.3390/ijerph192114092

**Published:** 2022-10-28

**Authors:** Masami Nakamoto, Norio Ideguchi, Satoru Iwata, Shunsuke Tomita, Nao Morimoto, Shion Fukuda, Shintarou Kudo

**Affiliations:** 1 Inclusive Medical Science Research Institute, Morinomiya University of Medical Sciences, 1-26-16, Nanko-kita, Suminoe-ku, Osaka 559-8611, Japan; 2 Department of Physical Therapy, Morinomiya University of Medical Sciences, 1-26-16, Nanko-kita, Suminoe-ku, Osaka 559-8611, Japan; 3 Graduate School of Health Science, Morinomiya University of Medical Sciences, 1-26-16, Nanko-kita, Suminoe-ku, Osaka 559-8611, Japan

**Keywords:** plantar assessment, Semmes–Weinstein monofilaments, validity, reliability, clinically usable index

## Abstract

There is no standard clinically adaptable criterion for assessing plantar sensation for pre- and post-intervention comparisons. Studies using Semmes–Weinstein monofilaments (SWMs) to investigate intervention effects on plantar sensation vary in procedure and do not consider measurement errors. This study aimed to develop a simple criterion using SWMs to assess plantar sensation, determine the measurement error range, and identify areas of low error. Six examiners assessed 87 healthy young adults in Experiment 1, while two examiners assessed 10 participants in Experiment 2. Filaments were graded from 1 to 20 based on increasing diameter. The smallest grade that could be perceived for three sequential stimuli was used as the criterion (smallest perceivable grade, SPG). The SPG was significantly smaller at the hallux and larger at the heel than at other sites. There were no significant differences between the SPG of the repeated tests performed by the same versus different examiners. The interquartile range of the differences was <±3 at all sites. Thus, our criteria were reliable in evaluating the effects of plantar sensation interventions, especially at the heel and the middle of the metatarsal heads and could contribute to the development of more effective treatments for plantar sensations.

## 1. Introduction

The primary role of plantar sensory function under loading conditions is to perceive changes in body movement and the environment [1,2,3]. Physiological and pathological aging related to this function increases the risk of falls [4]. Strong correlation between plantar sensation and static balance in hemiplegic patients has been previously suggested [5], and that stimulation of plantar sensation could improve static balance [6]. However, data on the therapeutic exercises that effectively improve this function remain limited. Thompson et al. have reported a training program for sensorimotor integration that was effective in improving balance, although it remains nebulous whether the sensation itself was improved [7]. In studies on interventions to improve plantar sensation and balance in patients with diabetes, the interventions were whole-body exercise (such as Tai Chi [8]), hence whether the mechanism is for improving plantar sensation is unclear. One reason as to why assessments for plantar sensation are unclear is attributed to the lack of widespread, clinically adaptable assessment methods for comparing plantar sensation before and after an intervention.

Clinically adaptable methods require inexpensive and easily accessible tools, along with short examination periods. Moreover, it is important to distinguish between interventional effects and measurement errors. This study focuses on the use of Semmes–Weinstein monofilaments (SWMs), which consist of 20 sets of filaments with different diameters, as a clinically adaptable method. The SWM was developed to identify the threshold of static tactile sensations of the hand [9]. For each monofilament, the force required to bend them was determined, thereby allowing for quantitative sensory testing. The SWM test is instrumental in assessing the degree of peripheral neuropathy damage attributed to a disease, such as diabetes [10] or surgery, as well as the recovery process [11]. SWMs have been employed to investigate the effects on plantar sensation before and after an intervention. However, the procedures used in different studies varied, with some lacking a detailed description [12]. Furthermore, the testing duration required to examine the sensory thresholds of multiple areas of the plantar foot in detail using a SWM is prolonged [13]. Negative reports on the reliability of SWMs are prevalent. For instance, Voerman et al. have reported that sensory thresholds differ from the generally proposed normal values, suggesting that the values may vary based on the procedure [14]. Chikai et al. have also suggested that the results obtained with SWMs could be affected by the hand movements of the examiner [15]. Conversely, the reliability of repeated testing of SWMs in the foot is moderate, when the examiner remains the same [16]. Therefore, for assessing plantar sensation before and after intervention using SWMs, simple criteria that can promptly identify plantar sensory characteristics with a known range of error for retests are warranted.

These criteria should be developed based on the characteristics of plantar sensation; previous studies have used procedures (or modified procedures) for the hand. The distribution of mechanoreceptors in the plantar region is rough compared to that in the palm of the hand [17]. The neural pathways are also longer than those of the hands. Functionally, the feet are not as conscious as the hands. Thus, a stimulus of a given magnitude applied to a specific site may or may not be perceptible, depending on the receptor type with receptive fields and whether the attention is focused on that site during stimulation. In fact, responses to small sensory stimuli in the plantar region are often ambiguous. Adopting a more perceptible stimulus intensity as a criterion for plantar sensation assessment should be considered [18] rather than the estimated threshold obtained by detailed examination, as previously indicated [19]. In addition, the distribution of mechanoreceptors in the plantar region suggests the presence of sites with small intra-individual variability, where the error of retest is small. Limiting the test site is an effective strategy in clinical practice.

This study aimed to develop a simple criterion using SWMs to assess plantar sensation, determine the measurement error range, and identify areas of low error.

## 2. Materials and Methods

This study consists of two experiments: Experiment 1 investigates the validity and simplicity of the criteria, while Experiment 2 investigates the testing error and reliability of the criteria.

### 2.1. Participants

Six examiners and 87 healthy young adults participated in Experiment 1, while two examiners assessed 10 participants in Experiment 2. The participants were students randomly selected from our institute. The participants were not aware of any neurological or orthopedic impairments that affected their daily lives. The characteristics of the participants are listed in Table 1. All examiners were trained on stimulation using the SWM and practiced thoroughly prior to experimentation. The practice session began with a skilled examiner acting as a participant and applying the filament perpendicularly to the skin over a period of 1.5 s. Initially, hand vibration may occur when applying the filament, hence practice was repeated until the skilled examiner felt a constant stimulus with the same filament.

Before participation, informed consent was obtained from the participants in verbal and written form. The study protocol was approved by the Ethics Committee for Clinical Investigation of Morinomiya University of Medical Sciences (Approval number: 2019-066). This study was carried out in accordance with the institutional and ethical guidelines for clinical research of the Japanese government and the Declaration of Helsinki. The data were anonymized, and confidentiality of information was assured.

### 2.2. Assessment Protocols of the Criterion Using SWMs

**Figure 1:**
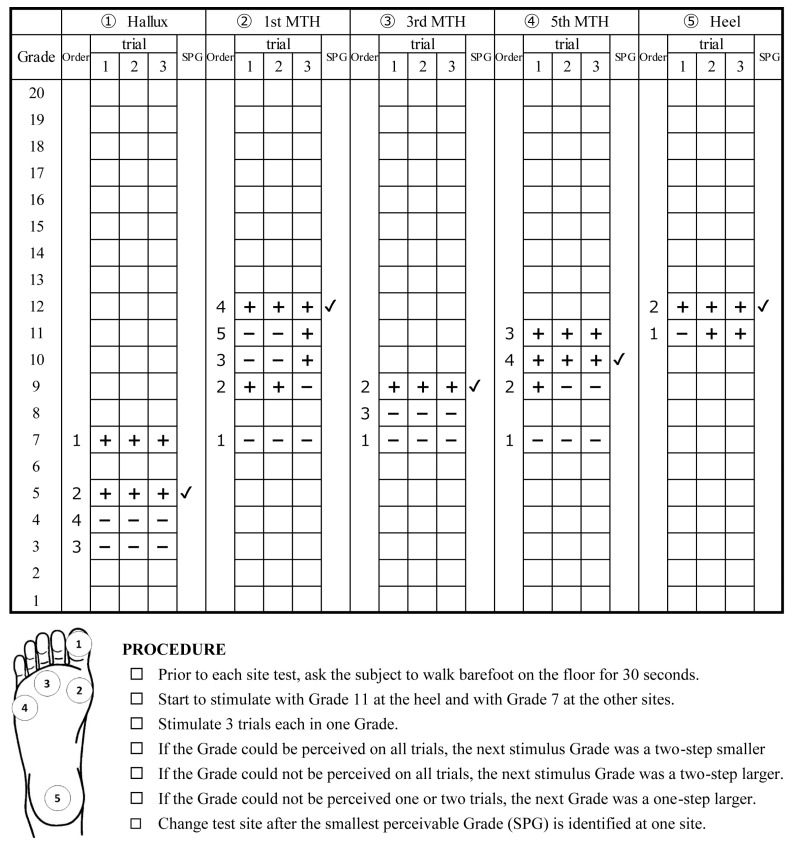
Representative records of a participant and the procedures and test sites of this study.

In this study, SWMs (Sakai Medical Co., Ltd., Tokyo, Japan) were defined as grades from 1 (No. 1.65) to 20 (No. 6.65) in order of decreasing diameter (Table 2), and the smallest grade that could be perceived for three sequential stimuli was used as the criterion (smallest perceivable grade; SPG). The tests were performed with the participant in the supine position with eyes closed in a quiet but not completely silent room, simulating a clinical rehabilitation scenario. Monofilaments were placed perpendicular to the skin, and pressure was applied for approximately 1.5 s until the filament forming a ‘C’ shape [20]. Participants were instructed to respond “yes” as soon as they felt the stimulus. The test sites included five regions on the right foot: the hallux; the 1st, 3rd, and 5th metatarsal heads (MTH); and the heel area, which were in contact with the ground in the standing position. The SPG at each site was identified using the following procedure (Figure 1). Using preliminary experiments and previous studies as reference [21,22], initially, we used a grade 11 (filament number 4.56) for the heel and grade 7 (filament number 3.84) for the other sites, which were stimulated three times. If the grade could be perceived in all three trials, the next stimulus grade was two sizes smaller; if the grade could not be perceived in all three trials, the next stimulus grade was two sizes larger. If the grade could not be perceived one or two times, the next grade was one size larger. The SPGs for each site were identified by repeating this procedure. It was possible to identify the SPG of one site with as few as nine stimulations, using three grades, within a few minutes of initiating the procedure. The order of the test sites was randomized between the participants. Before each site test, the participants were required to walk barefoot on a flat floor for approximately 30 s to reset plantar sensation and maintain constant conditions.

In Experiment 1, the time required to identify SPGs at the five test sites was recorded. Complaints of a residual stimulus sensation or response when no stimulus was applied were also assessed during the test.

In Experiment 2, an examiner identified SPGs at five test sites twice with a 15 min interval (intra-rater investigation). Different examiners performed the test randomly with a 15 min interval (inter-rater investigation). This interval was set to minimize the effects of temperature, humidity, and individual diurnal variation and to anticipate the expected intervention time in a clinical setting. The participants were instructed to wear socks and avoid prolonged walking and large impacts between the tests. During the test, the room temperature, humidity, and surface temperature of the test sites were monitored to ensure no drastic changes [23].

### 2.3. Statistical Analysis

No exclusion criteria were established, and all data were included. The comparison of the SPGs between each site (Experiment 1) and between the two tests (intra- and inter-rater investigation; Experiment 2) was performed using the values converted to logarithms of the force (g) that each filament can apply, as measured and labeled by the producer [16]. To assess the reliability of repetitive testing, the intraclass correlation coefficients (ICC) of the two tests (Experiment 2) were also calculated using logarithms [16]. Friedman and post hoc tests were performed to compare the SPGs according to the test site. The Wilcoxon signed-rank test was used to compare the SPGs tested twice by the same and different examiners. IBM SPSS Statistics version 28 (IBM, Chicago, IL, USA) was used for all the analyses. The significance level for all analyses was set at *p* < 0.05.

To facilitate future studies using our criteria, the median, interquartile range (IQR), and modes for each SPG site in Experiment 1 were obtained. To determine the magnitude of measurement error, the medians and IQRs of the differences between the SPGs identified by the two tests in Experiment 2 were obtained to determine the magnitude of the measurement error.

## 3. Results

### 3.1. Experiment 1

**Figure 2:**
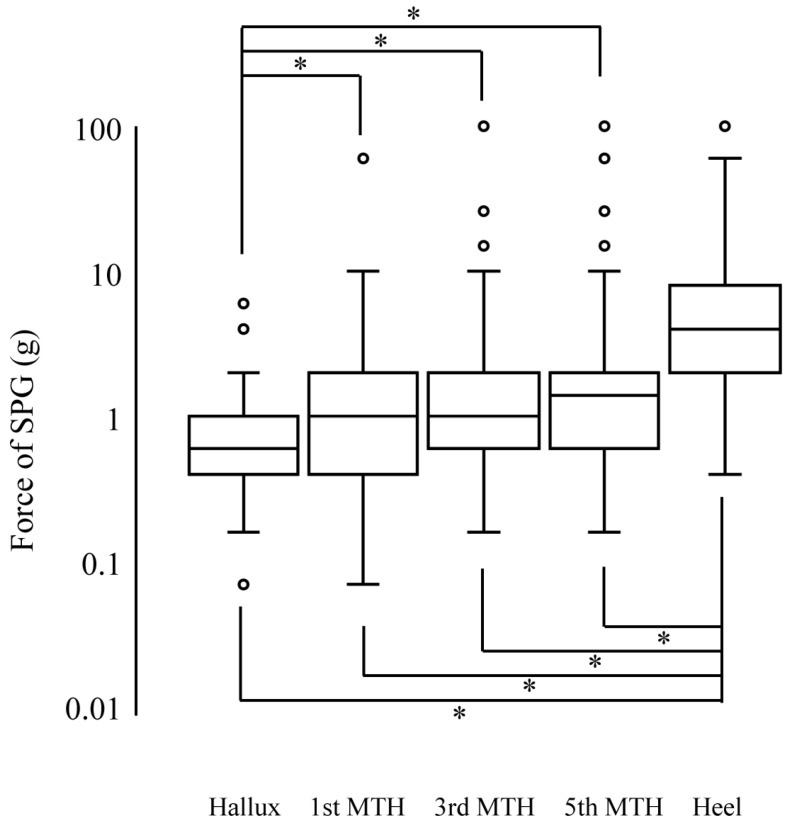
Comparison of the smallest perceivable grade (SPG) of healthy young adults at the hallux; 1st, 3rd, and 5th metatarsal heads (MTH), and heel. The SPG was converted to logarithms of the force (g) that each filament can apply, as measured and labeled by the producer. The symbol ° is indicates an outlier. *, *p* < 0.05.

The frequency distribution, median, IQR, and mode of the SPG for each test site are shown in Table 2. The medians for the hallux, 1st MTH, 3rd MTH, 5th MTH, and heel were 7, 8, 8, 9, and 11, respectively. A total of 11 of the 87 participants (13%) had SPGs of 14 or higher at several sites, except the hallux. The hallux had a significantly smaller SPG than the other sites, while the heel had a significantly larger SPG than the other sites (Figure 2). No significant differences were found in the MTH sites.

The average testing time was 13.7 ± 5.1 min. A total of 67 out of the 87 participants (77%) complained of residual stimulus sensation or responded when no stimulus was applied.

### 3.2. Experiment 2

No significant differences were observed between the SPG of the repeated tests performed by the same or different examiners. The intra- and inter-rater differences in the SPGs and their IQRs are shown in Table 3. The median values of the intra- and inter-rater differences were < 1 at all the sites, while the IQR of the differences was < ±3 at perceivable grade for each test site.

The ICCs of the intra- and inter-rater investigations and *p*-values are shown in Table 4. Significant correlations were obtained for the 1st MTH (0.53), 3rd MTH (0.82), and heel (0.64), considering the intra-rater investigation. Similarly, considering the inter-rater investigation, significant correlations were obtained for the 5th MTH (0.69) and heel (0.75).

## 4. Discussion

This study aimed to develop a clinically adaptable criteria for evaluating plantar sensation comparison before and after intervention. For the criteria, we considered that: (1) the method should use inexpensive, use easily accessible tools, and should not be time consuming, and (2) the range of error should be known. The “smallest SWM that could be perceived as three sequential stimuli” adopted in this study was the criterion that satisfied these conditions, was sufficiently adaptable in clinical practice, and has been discussed further in the following text.

### 4.1. Variability and Simplicity of the Criterion

In Experiment 1, SPG was obtained for each site in the 87 healthy young participants. The results demonstrated that the SPG was smaller for the hallux, followed by the MTHs and heels. The trend was similar to a previous study in which the SWM identified the plantar perceptual threshold in detail [21]. Jeng et al. have reported a higher threshold in the heel than forefoot sites, with no differences within the forefoot [22]. The criteria in this study were related to the perceptual thresholds obtained by the time-consuming procedure of the SWM.

Herein, the average testing time for the five sites was less than 15 min, which is considered acceptable for clinical applications regardless of the subjects’ mental fatigue. The reduction in testing time might be attributed to the fact that the criterion was identified by a simple procedure that allowed identification fewer times than the 4-2-1 stepping algorithm used in recent studies [21].

### 4.2. Reliability of the Criterion

Knowledge of the range of error and distinguishing it from the effect of the intervention is necessary for using this study criterion in clinical practice. Therefore, in Experiment 2, we analyzed the results upon testing by the same examiner (twice) and different examiners. The ICCs ranged from 0.41 to 0.82. Collins et al. [16] obtained ICCs for the 10 toes and medial and lateral feet with the hands procedure, reporting similar results. Since no significant differences were observed in the former results, no systematic errors owing to the consecutive presentation of the tests, such as habituation, were observed. In addition, no significant differences were observed in the latter result, suggesting the absence of systematic errors attributed to the examiners. Although systematic errors have been a challenge in previous studies of perceptual threshold determination [14,15], the exclusion of these influences was possible using this study criterion. Furthermore, the intra- and inter-rater differences between the two tests were smaller than 3. Thus, in an interventional experiment, if the criterion changes to a value more than 3 after a given intervention, it can be considered a significant difference. However, this judgment is possible only when the tests are conducted on the same day within a short period, as was the case in this study. If the two tests are performed on different days, various factors, such as life events immediately before the test [23] and body temperature [16,24], should also be considered. If necessary, the magnitude of the error, including this effect, should be investigated in future studies.

### 4.3. Useful Test Sites for Plantar Sensory Function

In this study, the test sites were selected from the plantar areas that contact the floor while standing and walking. In Experiment 2, the ICCs of the intra- and inter-rater investigations tended to be higher in the 3rd MTH and heel. In the standing posture, the load on the plantar surface is divided into two parts, the heel and the MTHs, wherein the greatest load is applied to the second and 3rd MTHs [25,26]. During normal walking, the heel and the middle of the MTHs are the paths of load transfer [27]. A high density of receptors is expected to be distributed in the areas where load changes occur [17,28]. As a result, greater reproducibility was observed under the heel and 3rd MTH than at other sites. Furthermore, this finding supports previous research suggesting that sensory input from the heel and middle MTH plays a vital role in the perception of anteroposterior translation of the center of foot pressure during standing and gait [1,3,29]. Therefore, as representative test sites for plantar sensory function, the heel area and the middle of the MTH were suggested to provide more reliable results and functional significance.

### 4.4. Precautions for the Use of the Criterion

Although all participants were not currently aware of any neurological or orthopedic impairments that affected their daily lives, 13% of the participants had one or more areas where filament No. 5.07, used in screening for diabetes [30], that was not perceivable. In the present study, we did not acquire medical history or conduct a detailed examination of potential diseases, they may have had potential diseases that could have caused their sensory impairment. Alternatively, these difficult-to-feel areas could be due to lifestyle habits such as walking style, type of footwear, or the type of sports played in the past. Additionally, approximately 80% of the participants complained of a residual stimulation sensation or responded to it, even though they were not stimulated in this study. However, this phenomenon disappeared immediately following the interruption in walking. This phenomenon may be attributed to the fact that the participants’ attention was being directed to areas where they would not normally pay attention. While this needs to be investigated in future studies, it is important to know that even healthy participants may have some areas of reduced or abnormal sensation or complaints in the test for plantar sensation.

### 4.5. Study Limitations

The criterion proposed in this study can be used not only as pre- and post-intervention evaluation but also for the early detection of a decline in plantar sensory function and even for preventing falls in older people [31]. To this end, it is necessary to collect results from various age and case groups in future studies.

This study has some limitations. The criteria in this study were identified in response to tactile stimuli and only observed perceptual and cognitive results processed by the cerebral cortex. Detailed studies of plantar sensory function are ongoing in the neurophysiological field [32,33,34]. The criteria of this study should be investigated in combination with these neurophysiological techniques in future studies.

## 5. Conclusions

Previous studies, using SWMs for evaluating the effect of an intervention on plantar sensation, have used time-consuming and various procedures, without considering measurement errors. Conversely, in this study, we set the criterion to the smallest filament that could be perceived as three sequential stimuli and investigated its validity and reliability. This criterion allowed one site to be assessed in >3 min; the magnitude of the measurement error was within three filaments. More reliable test sites to repeat testing are the heel and middle of the MTHs. Although it is necessary to collect data from various age and condition groups in future studies, this criterion is potentially applicable in clinical practice and will likely contribute to developing more effective treatments strategies for plantar sensations.

## Figures and Tables

**Table 1:** Characteristics of the participants.

	Experiment 1 (*n* =87)	Experiment 2 (*n* = 10)
Age (years)	20.7 ± 1.1	20.7 ± 0.5
Sex (*n*)		
Female	39 (44.8%)	6 (60.0%)
Male	48 (55.2%)	4 (40.0%)
Height (cm)	165.6 ± 8.7	161.8 ± 8.2
Weight (kg)	59.8 ± 10.2	54.3 ± 8.5
BMI (kg/m^2^)	21.7 ± 2.5	20.6 ± 1.9
Foot length (cm)	24.2 ± 1.6	24.0 ± 1.9

**Table 2:** Frequency distribution, median, interquartile range, and mode of the smallest perceivable grade for each test site.

Filament No.	Grade	Hallux	1st MTH	3rd MTH	5th MTH	Heel
6.65	20	0	0	0	0	0
6.45	19	0	0	0	0	0
6.10	18	0	0	1	1	1
5.88	17	0	3	0	1	2
5.46	16	0	0	2	1	1
5.18	15	0	0	2	1	2
5.07	14	0	2	1	3	11
4.93	13	0	0	2	1	5
4.74	12	2	7	3	6	15
4.56	11	2	8	3	7	23
4.31	10	6	9	10	18	13
4.17	9	11	7	14	13	7
4.08	8	11	10	13	10	3
3.84	7	17	15	20	12	3
3.61	6	22	20	13	10	1
3.22	5	15	5	3	3	0
2.83	4	1	1	0	0	0
2.44	3	0	0	0	0	0
2.36	2	0	0	0	0	0
1.65	1	0	0	0	0	0
Median Grade (IQR)		7 (6 to 8)	8 (6 to 10)	8 (7 to 10)	9 (7 to 10)	11 (10 to 13)
Mode of Grade		6	6	7	10	11

MTH: metatarsal head; IQR: interquartile range.

**Table 3:** Intra- and inter-rater differences of the smallest perceivable grades (SPGs) and their interquartile ranges (IQRs). Negative values indicate that the SPG in the first test was smaller in the intra-rater investigation and SPG of one examiner was smaller in the inter-rater investigation.

	Hallux	1st MTH	3rd MTH	5th MTH	Heel
Intra-rater difference	0.0	0.5	−0.5	0.0	−1.0
(IQR)	(−2.0 to 1.0)	(−2.0 to 2.0)	(−1.0 to 1.0)	(−1.3 to 1.3)	(−1.0 to 0.5)
Inter-rater difference	0.0	0.0	1.0	1.0	−0.5
(IQR)	(−1.3 to 1.0)	(−2.5 to 2)	(−1.3 to 2.3)	(−0.3 to 2.0)	(−2.0 to 0.5)

MTH: metatarsal head.

**Table 4:** Intraclass coefficients (ICCs) of intra- and inter-rater investigation and *p*-values.

	Hallux	1st MTH	3rd MTH	5th MTH	Heel
Intra-rater investigation					
ICC	0.41	0.53	0.82	0.50	0.64
*p*-value	0.093	0.038	0.000	0.053	0.013
Inter-rater investigation					
ICC	0.47	0.52	0.51	0.69	0.75
*p*-value	0.070	0.052	0.053	0.010	0.004

MTH: metatarsal head.
